# Acupuncture for Internet addiction: A protocol for systematic review

**DOI:** 10.1097/MD.0000000000024872

**Published:** 2021-03-26

**Authors:** Yalin Chen, Lingrui Zhang, Yan Liu, Yan Yang, Mimi Qiu, Yang Wang, Wei Peng, Hui Li, Tianmin Zhu

**Affiliations:** a School of Rehabilitation and Health Preservation, Chengdu University of Traditional Chinese Medicine, Chengdu; b Department of Medicine, Leshan Vocational and Technical College, Leshan; c School of Clinical Medicine; d School of Acupuncture and Tuina, Chengdu University of Traditional Chinese Medicine; e School of Medicine, Chengdu University, Chengdu, China.

**Keywords:** acupuncture, internet addiction, protocol, systematic review

## Abstract

**Background:**

Internet addiction (IA) has become a global problem characterized by excessive use of the internet, compulsive, and deleterious personal behaviors. Acupuncture has gained more and more attention in the treatment of IA. However, evidence of its effectiveness is lacking. The purpose of this systematic review is to evaluate the efficacy and safety of acupuncture in the treatment of IA.

**Methods:**

The following databases will be searched from the inception to September 30, 2020: the Cochrane Library, PubMed, Embase, Web of Science, China National Knowledge Infrastructure, Wan-fang database, Chinese Biomedical Literature Database, and Chinese Scientific Journal Database. The research on acupuncture and IA meets the screening criteria, and two independent reviewers performed citation screening, data extraction, and risk assessment of bias. We used Cochrane Review Manager 5.3 software for statistical analysis.

**Results:**

The findings will be published at scientific conferences or in a peer-reviewed journal. This study is based on the existing research, so there is no need for ethical approval.

**Conclusion:**

This systematic review provides evidence for the efficacy of acupuncture in treating IA disorder, and it is of great significance for effective clinical routine treatment of IA.

**Trial registration number:**

INPLASY 2020120099

## 1 Introduction

With the popularity of the internet worldwide, excessive use of the internet has become a common phenomenon, leading to the features of an “internet addiction (IA).”^[1,2]^ According to the reports in the past 10 years, the Philippines (51%) and Japan (48%) have the highest prevalence of IA.^[3]^ In China, 19% of people have problematic internet use.^[4]^ In Hong Kong, 35% of students have problematic internet use.^[5]^ IA, also known as pathological internet use, refers to excessive, compulsive, and improper use of the internet, which cannot focus on daily life.^[6–9]^ Although IA is not defined as a disease, controversial disagreements continue. In the latest version of the “Diagnostic and Statistical Manual of Mental Disorders,” internet gaming addiction is listed as an emerging disease worthy of further discussion.^[10]^

Currently, a variety of interventions have been used to treat IA, including cognitive-behavioral therapy, drug therapy, psychotherapy, and so on.^[11–13]^, acupuncture is an essential part of traditional Chinese medicine.^[14]^ Numerous study shows that acupuncture has a definite effect on addiction.^[15,16]^ Acupuncture is effective in relieving the neuropsychiatric symptoms of IA, such as anxiety,^[17,18]^ depression,^[18,19]^ and internet craving.^[20]^ However, there is no systematic review of the clinical trials of comprehensive acupuncture in IA treatment.

Our research reviews published studies on acupuncture treatment of IA in terms of research type, author, country, type of acupuncture, and treatment effectiveness, and provides a reference for acupuncture's clinical efficacy in treating IA.

## 2 Methods

This protocol has been registered on INPLASY, and the number is 2020120099. The system review protocol was implemented according to the systematic reviews and meta-analysis preferred report items guidelines (PRISMA-P).^[21]^

### 2.1 Eligibility criteria

#### 2.1.1 Types of studies

Inclusion criteria: (1) single-blind, double-blind, or non-blind design randomized controlled trial (RCTs) for evaluating IA's acupuncture treatment will be included. (2) Participants met the diagnostic criteria of IA and were assessed with Young's diagnostic questionnaire.^[22]^ (3) Participants who are diagnosed with IA (or problematic internet use and Internet addiction disorders) with other criteria.

Exclusion criteria: (1) non-randomized controlled trial, observational research, letters, editorials, case reports, conference, or abstract literature, were excluded. (2) The language of literature is not Chinese, English, or Korean.

#### 2.1.2 Types of participants

All participants were diagnosed with IA, patients with comorbid diseases whose primary diagnosis is IA will also be included, and participants were not restricted by age, gender, education, or race. Participants with significant depression, personality disorder, schizophrenia, and severe organic diseases were excluded.

#### 2.1.3 Types of interventions

Acupuncture treatment as the primary intervention method will be included, such as acupuncture treatment or acupuncture treatment combined with other treatment groups. There were no restrictions on the type of acupuncture, involving auricular acupuncture, traditional acupuncture, scalp acupuncture, electro-acupuncture, and so on. During the treatment process, the course, time, and frequency of acupuncture were no limitations. The control group was treated with placebo controls (sham acupuncture, sham interventions, or other therapy).

#### 2.1.4 Types of outcomes

##### 2.1.4.1 Primary outcomes

The primary efficacy endpoint was the improvement of internet craving (checked by Internet Addiction Test, IAT scale), self-control (SCS scale), compulsive (Yale-Brown Obsessive Compulsive Scale, YBOCS), anxiety (Self-Rating Anxiety, SAS), and depression (Hamilton Depression Rating Scale, HAMD), Self-Rating Depression Scale, SDS).

##### 2.1.4.2 Secondary outcomes

The secondary efficacy endpoint includes the average time spent on the internet (hours/day), impulsivity (Barratt Impulsiveness Scale-11, BIS-11), and assessment of scales related to the quality of life. Adverse events associated with acupuncture (eg, slight dizziness, nausea, and minor bleeding) or any other therapy.

To test the sustained effect of acupuncture treatment, we will evaluate the impact at the end of the follow-up.

### 2.2 Search strategy

The literature search will be identified by eight databases, the Cochrane Library, PubMed, Embase, Web of Science, and four Chinese databases, including Wan-fang database, China National Knowledge Infrastructure, Chinese Biomedical Literature Database, and Chinese Scientific Journal Database. We searched these databases from their inception in September 2020. Literature language will be excluded except English, Chinese, and Korean. The detailed search strategy is shown in Table 1. The same method will be applied to other databases, and the terms will be modified as needed.

**Table 1:** Web of science search strategy.

Number	Search terms
1	Addiction
2	Internet addiction
3	Pathological internet use
4	Problematic internet use
5	PIU
6	IA
7	IAD
8	or/1–7
9	Acupuncture
10	Acupuncture treatment
11	Acupuncture therapy
12	Electroacupuncture
13	Acupoint
14	Needle
15	Scalp acupuncture
16	Auricular acupuncture
17	Body acupuncture
18	Superficial needling
19	Transcutaneous electrical nerve stimulation
20	Acupressure
21	Thread embedding acupuncture
22	or/9–21
23	Clinical trials
24	Trial
25	Randomized controlled trial
26	Controlled clinic trial
27	RCT
28	Randomized
29	or/23–28
30	8, 22, and 29

IA = internet addiction, IAD = internet addiction disorders, PIU = problematic internet use, RCT = randomized controlled trial.

We also searched the CAM database to avoid omissions, a particular database for acupuncture RCTs.^[23]^ We will also search for ongoing experiments on the international experiment registration website.

### 2.3 Data collection and analysis

**Figure 1:**
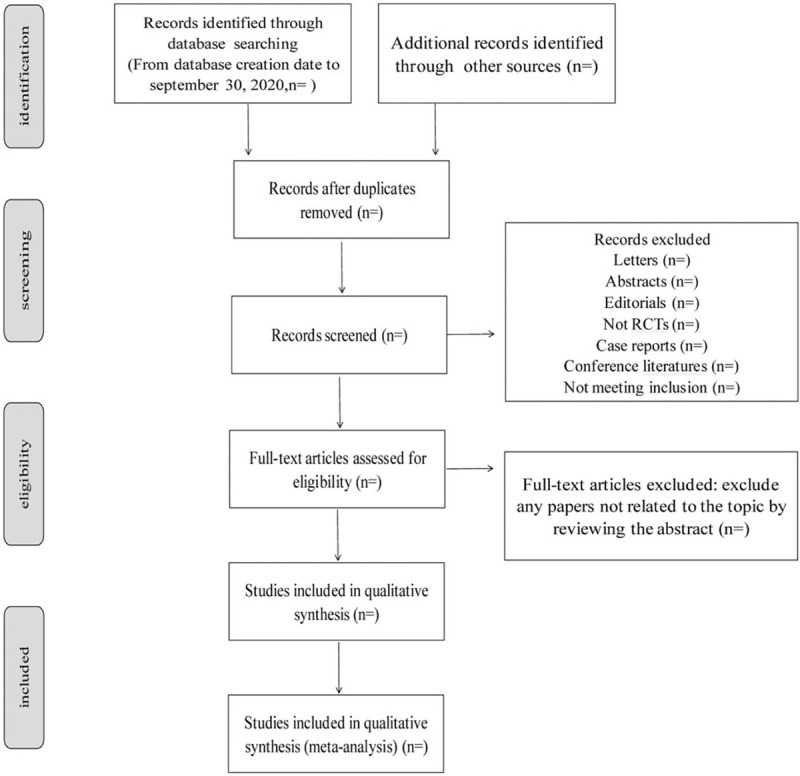
Flow chart of systematic review and meta-analysis of acupuncture treatment of internet addiction.

A pre-designed search strategy is used to search literature in the database. Two reviewers (YC and LZ) independently reviewed and evaluated the eligible literature. First, check whether the abstract and title meet the inclusion and exclusion criteria. Second, scan the full text to ensure compliance with inclusion criteria. Disagreements in screening were resolved through negotiation. If the conflict persists, it will be determined by a third reviewer. We will use endnote software to record the information and illustrate the detailed selection process in the PRISMA flowchart (Fig. 1)

#### 2.3.1 Data extraction and management

The two reviewers (YC and LZ) will independently extract data from previously screened literature. Then, the following information is extracted using a pre-designed data form: title, authors, year of publication, demographic characteristics, inclusion criteria, exclusion criteria, intervention methods of experimental group and control group, research design, diagnostic criteria, sample size, number, and frequency of acupuncture, duration of treatment and follow-up, outcome measurement, research results, and adverse events. If necessary, we will contact the author by email, and any disagreement will be resolved through discussion or consultation with the third reviewer.

#### 2.3.2 Assessment of risk of bias

Two reviewers (YL and YY) will assess the risk of bias using the Cochrane Collaboration tool.^[24]^ The content of the assessment includes: generation of random sequence (selection bias), allocation concealment (selection bias), blinding participants and personnel (performance bias), results blinding of evaluators (detection bias), incomplete result data (attrition bias), selective reporting (reporting bias) and other sources of bias, the risk of bias will be rated as “low risk,” “high risk,” or “unclear risk” in each area.

#### 2.3.3 Measures of treatment effect

The dichotomous data results were expressed as a risk ratio with 95% CI. For continuous data. We used mean difference or standardized mean difference to evaluate the effect of treatment with 95% CI.

#### 2.3.4 Dealing with missing data

When data is insufficient or absent. If necessary, we will contact the author of the original article for more information or clarification. If accurate data cannot be obtained, we will analyze the available data.

#### 2.3.5 Assessment of heterogeneity

We will use the *I*^2^ value (Higgins *I*-squared test) to detect the heterogeneity of research. The test of *I*^2^ ranges from 0% to 100% and is divided into 4 levels. *I*^2^ value >50% is considered significant heterogeneity.

#### 2.3.6 Data synthesis

All statistical analysis will be performed using Review Manager v.5.3 software. When the *I*^2^ value <50%, we will use the fixed-effect model to synthesize the data, random-effects model will be used for evaluation when *I*^2^ > 50%. When heterogeneity is significant and cannot be explained by clinical pathways, we will conduct a subgroup analysis.

#### 2.3.7 Subgroup analysis

We will conduct subgroup analysis to detect the sources of heterogeneity, including general information (age and sex), type of acupuncture (eg, auricular acupuncture, scalp acupuncture, and electro-acupuncture), course of treatment, type of control (placebo acupuncture, wait-list control, no therapy, or other treatment), results in measurement.

#### 2.3.8 Sensitivity analysis

We conduct sensitivity analysis to screen out high-risk bias and ensure the robustness of the results. Based on the missing data, sample size, and research characteristics, we will re-analyze and compare the original data's differences to evaluate the robustness.

#### 2.3.9 Ethics and dissemination

Since local ethical committees approved each eligible research, and the subjects have signed the informed consent, this study does not need further ethical approval. The results of this protocol will provide an effective and reliable basis for acupuncture treatment of IA, as well as enlightenment for clinical treatment of IA, and will be disseminated through peer-reviewed publications.

## 3 Discussion

This is the first systematic review on the effectiveness of acupuncture in the treatment of IA. Many studies have shown that acupuncture significantly affects addictive diseases and reduces the withdrawal/craving symptoms of substance addiction.^[14,25–26]^ Similarly, in IA, acupuncture has also been reported to reduce IA individuals’ craving, anxiety, and depression symptoms. Although previous studies have reported positive findings on acupuncture to treat IA, there is still insufficient evidence to support it.

The systematic protocol will provide the latest evidence for the effectiveness and safety of acupuncture treatment of IA. These findings have guiding significance for acupuncture's clinical application in treating IA and providing new treatment references for IA withdrawal institutions. This study's limitation is that the included literature is limited to English, Chinese, and Korean due to language barriers. Different types of acupuncture methods will affect the heterogeneity of acupuncture. Based on this systematic review, further experimental studies will be designed and improved.

### 3.1 Amendments

We will update our protocol if there are any changes during the research

## Author contributions

**Conceptualization:** Yalin Chen, Lingrui Zhang, Yan Liu.

**Data curation:** Yalin Chen, Hui Li.

**Formal analysis:** Yan Yang, Mimi Qiu.

**Funding acquisition:** Tianmin Zhu, Hui Li.

**Investigation:** Yalin Chen, Lingrui Zhang, Yan Liu, Yang Wang, Mimi Qiu, Wei Peng.

**Methodology:** Yalin Chen, Yang Wang, Wei Peng.

**Supervision:** Tianmin Zhu.

**Writing – original draft:** Yalin Chen, Lingrui Zhang.

**Writing – review & editing:** Hui Li, Tianmin Zhu.
